# A Review of Successes and Impeding Challenges of IoT-Based Insect Pest Detection Systems for Estimating Agroecosystem Health and Productivity of Cotton

**DOI:** 10.3390/s23084127

**Published:** 2023-04-20

**Authors:** Denis O. Kiobia, Canicius J. Mwitta, Kadeghe G. Fue, Jason M. Schmidt, David G. Riley, Glen C. Rains

**Affiliations:** 1 College of Engineering, University of Georgia, Tifton, GA 31793, USA; 2 Department of Agricultural Engineering, School of Engineering Science and Technology, Sokoine University of Agriculture, Morogoro P.O. Box 3003, Tanzania; 3 Department of Entomology, University of Georgia, Tifton, GA 31793, USA

**Keywords:** cotton pests, pest detection, pest control, machine learning, computer vision, precision agriculture

## Abstract

Using artificial intelligence (AI) and the IoT (Internet of Things) is a primary focus of applied engineering research to improve agricultural efficiency. This review paper summarizes the engagement of artificial intelligence models and IoT techniques in detecting, classifying, and counting cotton insect pests and corresponding beneficial insects. The effectiveness and limitations of AI and IoT techniques in various cotton agricultural settings were comprehensively reviewed. This review indicates that insects can be detected with an accuracy of between 70 and 98% using camera/microphone sensors and enhanced deep learning algorithms. However, despite the numerous pests and beneficial insects, only a few species were targeted for detection and classification by AI and IoT systems. Not surprisingly, due to the challenges of identifying immature and predatory insects, few studies have designed systems to detect and characterize them. The location of the insects, sufficient data size, concentrated insects on the image, and similarity in species appearance are major obstacles when implementing AI. Similarly, IoT is constrained by a lack of effective field distance between sensors when targeting insects according to their estimated population size. Based on this study, the number of pest species monitored by AI and IoT technologies should be increased while improving the system’s detection accuracy.

## 1. Introduction

In recent years, the most challenging initiative for pest management decisions has been assisting farmers in automated artificial intelligence sensor-based technologies and Internet of Things (IoT) applications. A lack of artificial intelligent sensor-based technologies and IoT leads to farmers attempting to scout the pests themselves, enlisting the help of extension specialists and crop consultants to reduce the losses associated with insect pest crop damage. The high crop loss rate (approx. 40%) due to pests may be a consequence of scouters’ inability to detect insect pest buildup in both production fields and nearby crops in real time [1,2,3,4]. There are many pests in the global cotton ecosystem (approx. 1000 species) [1], and nearly 125 per country [2]. When scouting such numerous field pests without sensors, some species may be practically impossible to detect with the naked eye [5]. The drawbacks of scouting without artificial intelligent sensor-based technologies and IoT applications include pesticide reapplication costs, pesticide resistance, health risks, and environmental-ecosystem pollution [6,7]. In addition, manual scouting may lead to a lack of focus on using selective pesticides and maintaining beneficial arthropods (predators and parasitoids) as biological control agents [8,9,10].

Therefore, this review paper focuses on the perspectives and opinions of researchers on the technical success and drawbacks of artificial intelligence and the Internet of Things when detecting, classifying, and counting cotton insect pests. Cotton growers have urged researchers to design Artificial Intelligence (AI) sensor-based systems and communication technologies to identify, classify, and monitor pests [9,11]. However, while many computing technologies have emerged, a review of the existing approaches’ current status, limitations, and pitfalls is needed to optimize future systems. For example, the use of AI technologies, such as the K-nearest neighbors (KNN), logistic regression, decision tree, support vector machine (SVM), and deep convolutional neural network (CNN) models may accurately and precisely help farmers to detect, classify insect pests and suggest the appropriate pesticides [12]. Such AI techniques are mostly recommended as certain insect pests are tiny and hard to detect [13]. Additionally, AI techniques are essential because of the potential to identify pests in images using low-cost RGB cameras [14]. However, few review studies reflect the perspectives and opinions of current research when designing/deploying AI approaches to detect, identify, and monitor insect pests, specifically in cotton fields.

## 2. Scope of the Review

This study was mainly confined to detecting and classifying insect pests in cotton settings. The articles that suggested predicting pest existence without detecting them were excluded from this study. Moreover, articles were not included except if detection involved an AI approach, as this study intended to understand the potential of AI, its associated challenges, and its success in the cotton environment. The existing literature was searched using the following keywords: “cotton pests”, “pest features”, “Artificial intelligence model in cotton pest detection”, “machine learning”, “IoT in the cotton field”, “cotton insect classification”, “cotton insect identification methods”, “cotton pest counting systems”, and “cotton pest image processing.” The literature associated with the keywords was identified in the database and indexed sources, including Google Scholar, Science Direct, connected papers, UGA Libraries Multi-Search, Web of Science, and Scopus. The study also used Google’s search engine and YouTube to collect information on the advertisement of related cotton pest detection systems, as the literature covering the intended topic was limited. The evaluation of the retrieved paper included the detected insect, data acquisition, utilized device, tested object detection and processing algorithm/AI models, performed activities (detection, classification, and counting) challenges, and system performance. The paper’s organization includes the following sections: Section 3 begins by understanding the common pest recognition features. Section 4 provides the commonly detected cotton pest species, approach, and performance indicators during the implementation of detection or classification. Section 5 details the AI models currently tested for detecting cotton pests and their corresponding successes and challenges. Section 6 discusses manufactured intelligent systems/devices for identification, classification, and counting after detection and classification. This section also describes typical sensors and traps involved in monitoring cotton pests in the field. Section 7 shows AI in beneficial insects (predators and parasitoids) for controlling cotton pests or assisting in flower pollination. Section 8 discusses the challenges of implementing IoT devices. Section 9 reveals the urgent need for IoT applications in managing major cotton pests, and Section 10 provides recommendations for further research.

## 3. Pest Recognition Features

Obtaining the proper pest features is one of the essential prerequisites for effective pest recognition. The most prevalent strategy has been grouping the pixels in images of similar characteristics (image segmentation). The color distribution, morphology, texture (entropy properties), and local characteristics are some of the features that may be retrieved during the image segmentation process [15,16]. Color distribution is crucial during insect classification since different species of insects and their body parts come in multiple colors. In comparing and understanding the color of a specific insect pest, color indexing is often used to compare the color of retrieved images with that of the query [17]. The techniques to extract color features include a color histogram [14], color moments [18], and a color correlogram [19]. According to [17], the histogram technique has been a potential image signature due to its good accuracy and sensitivity regarding pest position and orientation dynamics. However, the color histogram technique has a limited ability to differentiate the spatial relationship between color patches. In capturing the spatial relationship of color patches and the entire color distribution, color correlograms and color coherence vectors have been proposed, primarily when used together. The utilization of color moments has been effectively employed in content-based image extraction systems and has been reported to be far more reliable in defining color distributions than the histogram technique [20].

The morphology and local features include contour length, boundary diameter, area, curvature, and perimeter. In contrast, local features involve the form factor, roundness, aspect ratio, compactness, and extension [21,22], as shown in Table 1. To be considered a key feature, it must have a low degree of association with other categories, have a more considerable relevance when seen by humans, and represent a significant variation compared to other insect categories. On the other hand, the analysis of textural properties involves contrast, correlation, entropy, energy, and homogeneity properties, especially utilizing the image gray-level matrix [23].

When distinguishing one insect pest from another, it is critical to choose the crucial criteria carefully to avoid using features that are not necessary, which can lead to complicated algorithms.

## 4. Detected Cotton Pest Species, Approach, and Performance Indicator

### 4.1. AI Performance Indicators

The performance of AI models such as Convolutional Neural Networks (CNN), Support Vector Machines (SVM), K-Nearest Neighbors (KNN), Artificial Neural Networks (ANN), RNN (Recurrent Neural Networks), DBN (Deep Belief Networks), and DBM (Deep Boltzmann Machine) is dependent on the sensor’s data clarity. The data collected by sensors and then processed using artificial intelligence models must be accurate to provide meaningful information [24]; the best accuracy depends on good sensor calibration before data collection [25]. As demonstrated in Table 2, the performance of artificial intelligence models is commonly tested for accuracy (%), precision, recall (sensitivity), and F-scores [12,21].

### 4.2. Detected Cotton Pest Species

The names of the cotton pests were identified in various research using AI image processing techniques, as shown in Table 3. We found that some pests, such as Cotton Whiteflies, Spiders, Pink bollworms, and American bollworms, were proven to be detected using AI models in multiple studies. Other pest species were only examined once in a single study. The choice to include a particular pest in this study may be based on the availability of image data to meet the model’s requirements, which are chosen for that particular study.

## 5. Tested AI Models for Pest Detection

**Figure 1:**
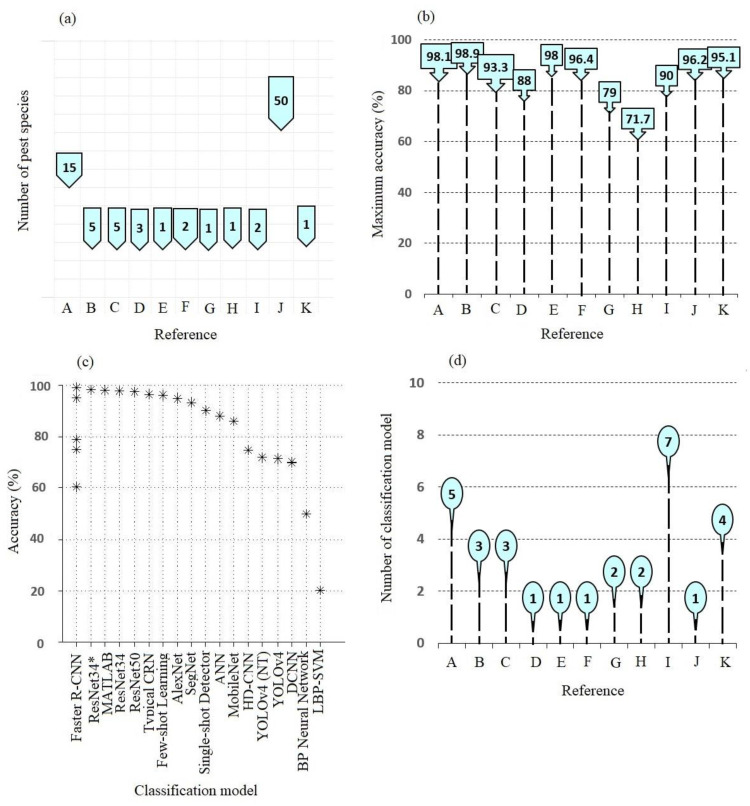
Summary of AI image processing algorithms used for detecting cotton pests. (**a**) The number of cotton pest species included in the study. (**b**) The highest accuracy reported in the study. (**c**) Comparison of accuracy of image classification models reported in cotton pest detection studies, and (**d**) = the number of classification models involved in an individual study. The references A, B, C, D, E, F, G, H, I, J, and K represent references [1,11,14,21,23,27,28,29,30,31,32], respectively. The (*) indicates improved ResNet34.

The studies of artificial intelligence (AI) for image-based cotton pest detection are shown in Figure 1. Although it is estimated that there are more than 1000 pests in the cotton environment, very few have been identified using AI (Figure 1a). However, using AI to identify cotton pests is promising based on best-reported accuracy in several studies (Figure 1b). According to our research, the range (71.7–98.9%) was the most frequently reported high detection accuracy across numerous investigations. Figure 1c displays a comparison of widely mentioned accuracy classification algorithms/models. Multiple studies investigated Faster R-CNN extensively and found it reasonably accurate. Although the studies were independent, typical CNN, Few-Shot Learning, and Single Shot Detector improved ResNet34 (ResNet34∗), SegNet, ANN, and MATLAB classifiers, which demonstrated a reasonable detection accuracy in cotton pest image classification. Furthermore, it was found that these effective image classification models were reported primarily by comparing multiple models (Figure 1d). Most of the research used upgraded CNN (Few-Shot Learning, Single Shot Detector, ResNet34, and SegNet) to attain maximum accuracy.

For example, using CNN models, fifteen (15) cotton insect pests were classified utilizing 100 original RGB color images and 620 augmented images for each insect [1]. The study controlled the augmentation process by rotating the original images in 10-degree increments and performing random sliding, stretching, and zooming up to 1.5×. The ability of the four CNN models: AlexNet, ResNet34, ResNet50, and ResNet34∗, were tested. ResNet34∗ was a significant attempt and contribution made by the authors to improve ResNet34 and, specifically, to attain a higher accuracy in cotton pest classifications. The CNN models were compared to the LBP-SVM model, which was developed using Linear Binary Features (LBP) and a Support Vector Machine for the baseline comparison. Although all of the CNN models in this study had better accuracy, the author claimed that the CNN models with residual architectures (AlexNet, ResNet34, and ResNet50) had more accuracy than the typical CNN model (AlexNet). Furthermore, the improved ResNet34 (ResNet34∗) demonstrated greater accuracy than the other evaluated models. The achievement of improving the ResNet34 models indicated the potential that the original CNN models might be modified for improved identification of cotton insect pests. One of the most significant challenges identified in this study was the models’ inability to classify images of insect pests with similar appearances, such as Heliothis virences (adult) and Spodoptera frugiperda (adult). Such a barrier may emphasize the researchers’ need to collect more data during detection rather than relying solely on images, as revealed in [33,34,35,36,37].

The CNN technique, called the “few-shot learning approach”, was developed to detect and learn cotton pests using a few images instead of many images as an input data set [24]. The goal of upgrading common CNN deep learning structures was to minimize the image processing time and effort spent gathering a big data set and applying high-powered or extensive field-hardware computing resources such as GPUs and TPUs during processing. Additionally, the authors wanted to avoid existing image pre-processing procedures such as target cropping, grey transformation, and resizing [38]. The suggested AI algorithm was also useful since, unlike GPUs, TPUs, and servers, it is compatible with low-cost embedded hardware that may be used in the field. After comparison, the authors found that the few-shot model outperformed other standard models such as GoogleNet, AlexNet, VGG-16, VGG-19, ResNet-50, and ResNet-101 when classifying cotton pests. This indicated that when embedded in running devices, the few-shot learning model may be considered one of the significant successes and promising techniques in image processing. This is because, when addressing some of the shortcomings of existing models, such as using a few training images, the need for a robust model training machine/computer results in the need to reduce data collection and model training time. Similar systems that swiftly compare the metric space between characteristics of input images have been proposed in Siamese network relation networks and prototype networks [39,40]. Despite an improved accuracy, the focus was on improving total model performance through a parallelism technique, which supports partitioning them into simple tasks and performing them simultaneously and accurately.

A comparison was conducted between a Deep Convolutional Neural Network (DCNN), Hierarchical Deep Convolutional Neural Network (HD-CNN), and Pixel-wise Semantic Segmentation Network (SegNet) to detect five cotton pests [14]. The authors intended to modify the SegNet and compare its performance with the typical (DCNN) and (HD-CNN) as a baseline. The performance accuracy of the SegNet model in identifying and classifying the five tested cotton pest insects was reported to overtake (DCNN) and (HD-CNN). They used pixel-wise classes that resulted in lower errors than the other approaches. Such a performance aligned with the need to merge the improved model with a robot that could save the obtained data set images, detect pests, produce and record coordinates using geographical navigation satellite systems (GNSS), and undertake insect pest mapping. More information on the influence of the image background, specifically during the separation of the image background and the targeted insect, is still an issue to investigate further. Furthermore, the authors expressed the need to choose the optimal number of automated convolutional layers optimizing training pace and model performance.

An application that includes an Internet of Things (IoT) device and a faster region-based convolutional neural network (Faster R-CNN) to classify insect pests through cloud computing was reported in [11]. This application utilizes a deep-learning model to send images of unknown crop pests to the cloud via an iOS or Android-based smartphone for storage, identification, and classification. The Faster R-CNN was quicker than previous image classification techniques such as the Single-Shot Multi-Box Detector (SSD) MobileNet and backpropagation (BP) neural networks. The accuracy (98.9%) of this suggested technique exceeded that of SSD MobileNet (86%) and the BP neural network (50%). The following are some of the promising contributions of the proposed model: detecting insect pests on complex visual backgrounds, recognizing pests in real-time, and forecasting agricultural pest classes and locations using the RPN module (Region Proposal Network).

Furthermore, the suggested mobile application and cloud computing were significant since they added the capability of proposing appropriate pesticides after the pest was classified. However, the number of pests examined (five) was insufficient. There is a need for future research to study a similar system with an increased number of cotton pests. Moreover, the effect of different mobile phone resolutions, varying illumination as crop growth conditions, and seasonal weather may need to be sufficiently investigated.

In a similar study, 120 images were used to compare the performance of Fast R-CNN and YOLOv4 detection models [32]. The Faster-RCNN model had a better accuracy of 95.08% (F-1 Score: 0.96, recall: 98.69%) compared to a YOLOv4 model that indicated a lower accuracy of 71.77% (F-1 score: 0.83, recall: 73.31%). However, the YOLOv4 model was faster than the Faster R-CNN.

Moreover, a CNN architecture was shown to identify and classify spider mites and leaf miners by collecting 600 RGB-colored images of insect pests [27]. The authors utilized the K-fold confirmation method to divide and upgrade the CNN model. The authors accomplished the deep learning process by combining Keras, TensorFlow, and Jupyter deep learning libraries. The developed model was 96.4% accurate for the tested classes after 100 epochs compared to 50 or 150 epochs. However, compared to the study by [1], which required 100 original images and 620 augmented images per insect category to achieve 98.1% accuracy using ResNet34∗, the work in [27] utilized substantially fewer images (600) to achieve 96.4% accuracy. This study also presented similar challenges, including complexity in the insect’s features (shape), dynamics due to light intensity, insect orientation, various backgrounds, and similarity in insect size.

In 2000 a Deep Neural Network was used to classify and differentiate insects in the cotton ecosystem [21]. The insect pests were classified using the decision tree method. The study involved eight (8) extraction features, including insects with and without legs. One of the most critical findings of this study was that the model could not accurately recognize all the insect pests. Four insect species were detected at 77.0, 85.7, 91.5, and 95.6% accuracy. According to the author, one of the reasons for poor classification was the high light reflectance of some insect pests. Despite providing a framework for cotton insect classification, the findings were not effectively applied or expanded until the expansion of deep learning studies in 2006 due to limitations in the computer system and neural network theory during that period [41,42]. The studies involving basic Deep Neural Networks were limited.

## 6. Intelligent Sensor Systems for Monitoring and Counting Cotton Pests

### 6.1. System Components of Remote Monitoring Devices

**Figure 2:**
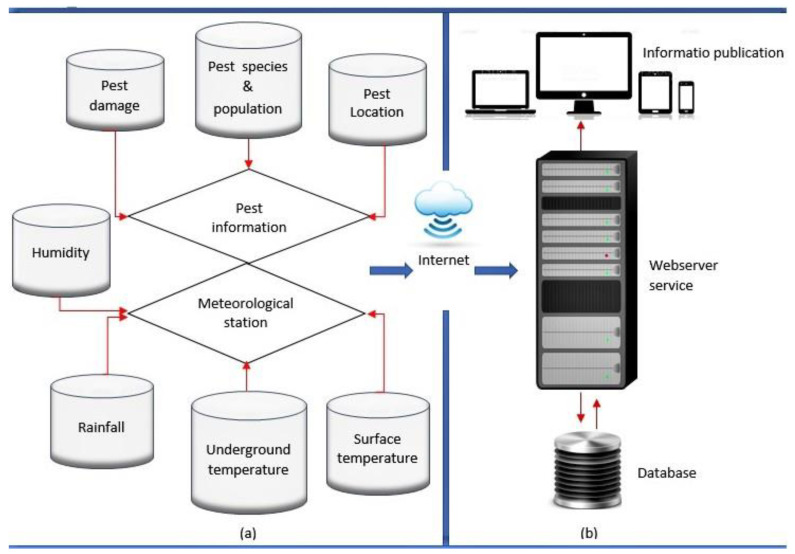
The components of the insect pest monitoring platform. (**a**) System for information collection, and (**b**) System for information analysis and display (adapted from [43]).

Remote monitoring platforms may include data collection, storage, analysis, and information dissemination [43]. The information-gathering system can incorporate several types of sensors, such as RGB, infrared, and hyperspectral cameras, to acquire images of insect pests. The resolution should be considered when choosing a camera, as a low resolution may negatively affect the image processing output [44]. Furthermore, environmental data, such as light, rainfall, soil moisture content, and underground and surface temperature sensors, may also be included in the information-gathering system for immediate pest detection and the prediction of population changes [43]. After collecting and handling data, data analysis was required to create valuable information that could be shared with users to alert them to take pest management action. Such information should be integrated with location information using GNNS to allocate the field site under pest damage or pollination [9]. With artificial intelligence models, the analysis section should be integrated with robust machine learning models that can immediately correlate the sensor data and provide pest management recommendations. For advanced pest tracking systems, the platform should provide multiple sensor data and work in two ways: it should deliver field information to the user and allow the user to query it at any moment, as illustrated in Figure 2.

**Figure 3:**
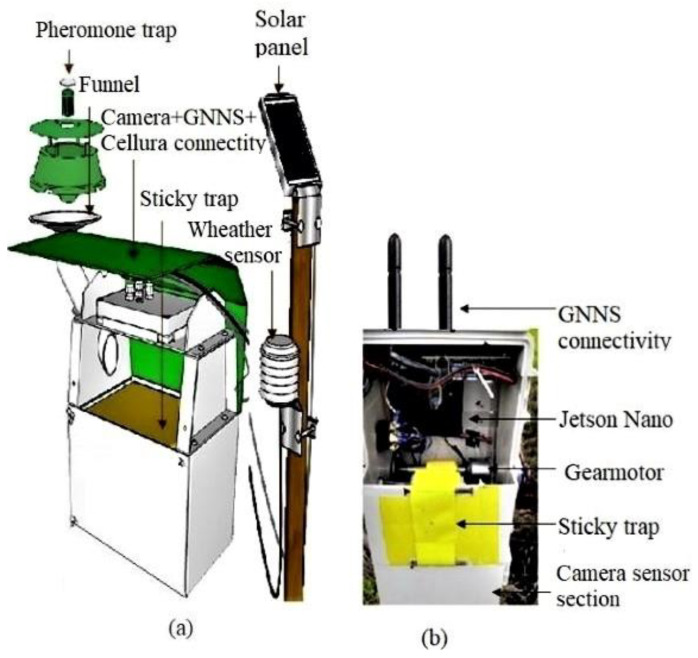
Remote monitoring devices for cotton pest identification. (**a**) Self-cleaning trap [45], also shown at https://app.efos.si/trapview_help/html/Trapview_SCM.html (accessed on 22 November 2022). Permission granted, and (**b**) Remote whitefly monitor [32].

In cotton fields, the systems to detect flying insects in cotton fields named the “self-cleaning trap” (Figure 3a) and “remote whitefly monitor” (Figure 3b) were shown in [32] and [45], respectively. The “remote whitefly monitor” and “self-cleaning trap” use sticky traps to fix the insects to the camera sensor’s position. However, in the “remote whitefly monitor”, the attractive mechanism of pests to the camera is the sticky trap (yellow, blue, or white color) itself, while in the “self-cleaning trap”, the pests are attracted to the camera position using pheromone. In addition, the whiteflies monitor device includes a function for counting pests and reporting online one to three times a day. Both devices have the potential for further improvement, particularly in attracting multiple pests and conducting specific insect classifications. These devices can also potentially include models that reasonably forecast pest dynamics, including weather conditions.

### 6.2. Counting of Pests on Leaves

Capturing pest images directly from leaves may avoid the cost of purchasing insect pests’ attractants, such as yellow sticky traps or pheromone agents. The technique for identifying and estimating whiteflies’ population directly from the cotton leaf was demonstrated [23]. This approach utilized MATLAB field-based machine learning techniques. The data collection and analysis pipeline included image acquisition, the conversion of RGB images to HSV (Hue, saturation, value), background removal, grey color thresholding, and whitefly counting using a bounding box and region props algorithm. The proposed technique for pest counting was both quick and cost-effective. Additionally, the method was robust, indicating an accuracy rate of 98%. This approach should also output the number of pixels, ratios, interior density, standard deviation, skew, and kurtosis in the event of multiple species detection after the feature extraction procedure [1]. However, image processing for whitefly recognition and counting in the field is still loaded with difficulties, especially in dust, excess moisture, weather conditions, and leaf veins that often indicate a light color similar to whiteflies. Moreover, because whiteflies are smaller, detecting and analyzing them becomes more challenging, mainly when individuals stick to each other [46]. In addition to insects’ small adhesion size, image processing becomes more complicated when the captured cotton image has poor quality [47].

Another study included transferring machine vision models to a mobile device App for agronomists to acquire images, analyze, and count Silverleaf whitefly nymphs on the cotton leaf [30]. The method used segmentation and machine learning. Silverleaf whitefly nymphs were recognized with up to 67% and 79% accuracy using segmentation-based and Faster R-CNN -learning approaches, respectively. More work is still being performed to make the device suitable for aphids and mites in cotton fields. The suggested device development process was reported in [31]. Smartphones from Apple, Sony, and Samsung were tested as part of the concept testing. The updates indicated that the greatest F-scores of 71.7 to 75.8% were found in deep learning models that used the iPhone alone or in conjunction with other smartphone models. All deep learning models that included Sony and Samsung combined or separately without integrating the iPhone model were reported with lower F-scores ranging from 44.0 to 55.6%. The model’s performance on the tested mobile smartphone missed the specified F-score mark from 90 to 95%. The inability to achieve the necessary F-score threshold was related to reduced image quality from using only one image sensor for training, detecting, and assessing performance in a real-world cotton field scenario.

Similarly, the classification of pests on plant leaves based on a smartphone running iOS 13.5 through cloud computing techniques, the Internet of Things (IoT), and a faster region-based convolutional neural network was demonstrated [11,30]. However, the complexity of the resemblance between the plant’s leaf backgrounds and some pests was still challenging. In encountering some of these problems, the utilization of high-resolution digital devices is essential though it is expensive to purchase them. The primary and minor axis lengths and eccentricity are widely employed to eliminate leaf veins from images [48].

### 6.3. Counting Pests on Sticky Traps

**Figure 4:**
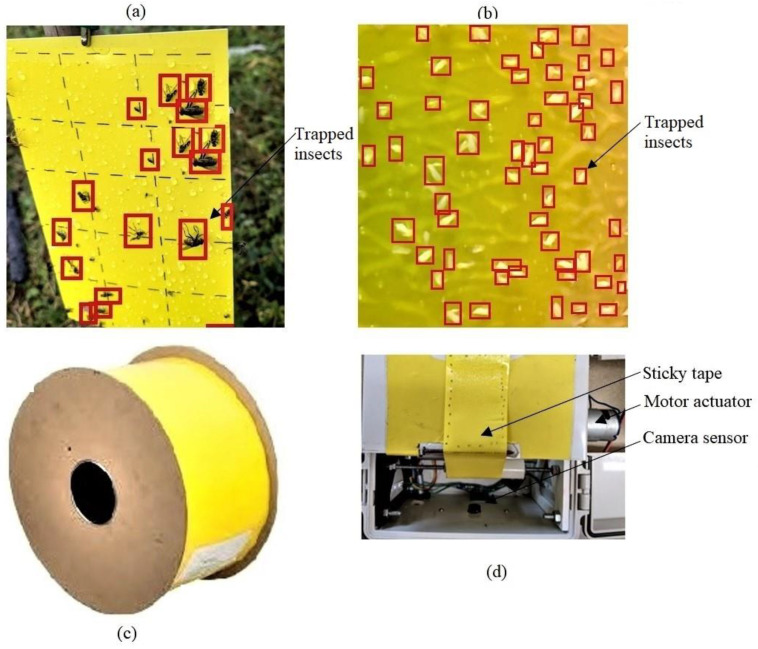
Photographing insect pests on the stationery and automated rotating sticky trap. (**a**) Pest detected on a non-automatic rotating sticky trap card, (**b**) Insects detected on the rotating sticky roller, (**c**) Sticky trap roll for an automatic rotating sticky trap, (**d**) Sticky trap under the automated rotating system (adapted from [32,50].

The use of sticky traps is essential in minimizing detection challenges between complicated backgrounds and the targeted insects. The sticky traps assist in sticking and fixing the insects while waiting for the image [49]. The procedure for automatically estimating the number of cotton pests, particularly whiteflies, using sticky traps is broadly suggested in [23,32,50]. The method may include image acquisition, color space conversion, background subtraction, thresholding operations, and morphological tracking/labeling operations. A camera may scan the insects on the non-automatically rotating sticky trap [50] as expressed (Figure 4a) or by automatically rotating the sticky trap roll [32] as shown (Figure 4b–d). Additionally, insect feature extraction from sticky tape may include training the model with labeled insect features using box boundaries or a background removal approach. The technique for automatically rotating the yellow stick roller in the morning and afternoon and shooting the trapped cotton pests was also demonstrated [32]. The proposed system could detect pests on yellow sticky tape, send images to a web server, count the pests, and send the insect count status to a web server. This study demonstrated the possibility of employing the Faster-RCNN detection and counting model, embedded computers, cameras, and sticky tape to identify and count real-time cotton pests. This system could potentially submit daily pest monitoring reports from multiple remote stations. Since the remote devices operate on solar energy and low-cost batteries, the technology may be feasible for field operations, particularly in developing countries. Automating rotating yellow tape may also be advantageous owing to minimal maintenance, particularly in lowering the time required for the field replacement of fixed stick traps. However, the training of the reported system relies on detecting and counting only whiteflies. Improving such a system to detect and classify multiple insect pests could significantly contribute to pest management. Moreover, since such devices may operate autonomously in the field, integrating other characteristics, such as micro-climates, may aid farmers in forecasting insect dynamics.

### 6.4. Counting Pests on Paper

This approach collects the trapped insects onto a sheet of paper for imaging. The trapping of insects involves attracting agents such as pheromones or light. Using a smartphone camera, a system that takes images of insects trapped by pheromones was studied [28]. The system processed the images and counted the pests using a Single Shot Multi-Task Detector (SSD) supervised Neural Network algorithm. The technique had the added benefit of indicating if the insect populations warranted spraying with an insecticide. Due to agro-climatic zones, the study acknowledged and addressed the issues of using low-resolution cameras and data diversity in cotton fields. This system, however, was only taught to detect a few pests, including pink and cotton bollworms. Because the system relies on human operation and the usage of mobile phones, human error and differences in the versions of mobile phone cameras might cause variances in the system’s performance. One strategy to avoid the complications of a smartphone camera has been to kill the pest in the trap without crushing it before acquiring the image. Such a practice may need a trained and committed farmer or consultant. In addition, the optimal number of traps to capture pests per area was not explicitly mentioned for a farmer to apply a pest management solution.

## 7. Artificial Intelligence in Beneficial Insects

The perspective of detecting or identifying beneficial insects involves crop pollinators (e.g., wild/honeybee and butterfly), predators (e.g., *Chrysoperla externa* Hagen, *Eriopis connexa* (Germar), *Podisus nigrispinus* (Dallas) and *Orius insidiosus* (Say)) [8,51], or parasitoids (e.g., *Aphelinus gossypii* (Timberlake), *Bracon vulgaris* (Ashmead), *Lysiphlebus testaceipes* (Cresson), *Telenomus podisi* (Ashmead), and *Trichogramma pretiosum* (Riley)) [51]. Detection and classification were conducted based on images of morphological features [44,52] or insect-swarming activities [53,54,55]. For example, when using bee morphological features with more than 9000 images under deep convolutional networks (CNN), the species and subspecies of wild bees were automatically recognized with accuracy rates of 99.3% and 98.05%, respectively [44]. Such accuracy findings were approximately similar to 98.5% reported in [56] when classifying and counting the Hymenoptera parasitoids of Aphelinidae, Braconidae, and Aphidiinae using Mask R-CNN. However, some classifiers did not perform better on the morphological features of beneficial insects. For example, poor accuracy levels (max. 65.15%) of bee’s morphological classifications were reported using Naive Bayes, Linear Discriminant Analysis (LDA), Logistic, Support Vector Machine (SVM), Multilayer Perceptron (MLP), KNN, and Decision Tree (C4.5) classifiers [52]. The capacity of deep CNN algorithms to automatically extract and learn traced or manipulated features straight from the data could be credited with increased accuracy when utilizing them [57]. The downside of this approach is that it needs many images or insect labels to achieve a satisfactory performance [43]. For example, over 9000 images and over 15,000 insect labels were utilized in [44,56] to achieve over 90% higher accuracy.

On the other hand, using acoustics is an alternative to the morphological feature extraction technique. Deep learning and IoT-based approaches for monitoring and categorizing bees based on the sound frequencies of their swarm activity were demonstrated [53]. While the compressed audio (MP3) lost 10% accuracy, the uncompressed audio showed a greater accuracy of almost 94%. By utilizing an embedded computer, the study had the potential to pinpoint where beehives were located. One of the challenges was delivering audio data continuously due to the beehives’ remote location. Creating MP3 files solved this problem by taking full advantage of the optimum cellular network bandwidth (16–64 kbps) for a few kilobits per second rather than 256 kbps for the uncompressed audio file. A similar acoustic machine-learning approach included additional features for forecasting and mapping the pollinated crop field area [9]. The sound frequencies were grouped into flying, fanning, hissing, and pipping at 250 Hz, 225–285 Hz, 3000 Hz, and 340–450 Hz, respectively. The analogy detection filter removed the audio fragments before sending them to the cloud for SVM processing. However, achieving an optimal number of microphone sensors per m^2^ and the proper distance between the microphone sensor and the flying insect remains challenging. For training and testing, the technique may also necessitate collecting massive amounts of audio recording data on swarm activities [54,55]. The option for obtaining suitable and sufficient sound recordings was to use open-source data [53]. The same acoustic detection approach was developed for flying insects such as bee pollinators [28]. The developed system included other field variables such as light, rainfall, soil moisture content, ambient temperature, surface temperature, and subsurface temperature. The system combined a raspberry pi processing unit, a raspberry pi camera, a YOLO object detection model, and a data server for monitoring flying insects. However, unless paired with a Support Vector Machine (SVM), the authors found the YOLO detection model’s capabilities in classifying flying insects limited. Unfortunately, studies that detect, classify, and quantify beneficial insect species using deep learning techniques, especially predators, are still limited.

## 8. Challenges to the Implementation of IoT-Filled Devices

The challenges that remote devices for pest detection in the field may encounter include high power consumption, network issues, inadequate security, service expiration, physical hardware defects, software failure, and changes in ambient conditions, as demonstrated in Table 4. The origin of a particular challenge on a device may change occasionally and depend on the design of that device. If necessary, the suitable operating IoT device installed on-site should be characterized by high performance with minor faults. In the process of avoiding drawbacks for field devices, Table 4 also shows the proposed solutions of different studies.

## 9. Urgent Need for IoT towards Worldwide Major Cotton Pests

**Figure 5:**
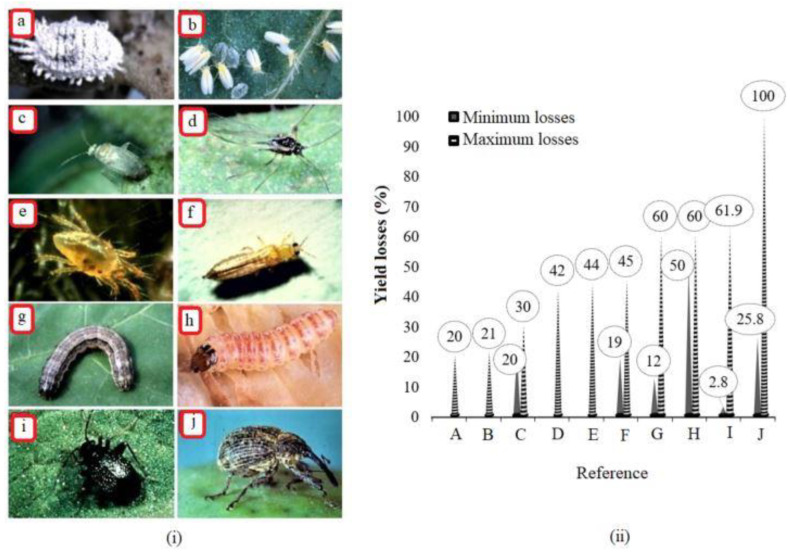
Serious pests in cotton fields (**i**) and corresponding yield lost per pest category (**ii**). In (**i**), a: mealybug, b: silverleaf whitefly, c: cotton leafhopper (adult), d: cotton aphid, e: two-spotted spider mite (adult), f: cotton thrips, g: fall armyworm (larva), h: pink bollwormi, i: fleahopper, and j: boll weevil (also shown at https://www.insectimages.org/ (accessed on 11 December 2022)). In (**ii**), A: Boll weevil [73], B: Cotton Thrips [78], C: Spider Mites [81,82], D: Fleahopper [74], E: Cotton aphid [79,80], F: Leaf hopper [83,84,85,86], G: Cotton mealybug [72,89,90,91], H: Whitefly [92,93,94], I: Pink bollworm [95,96], and J: Armyworm [75,76].

This study found that sensor-based artificial intelligence and IoT may need to devote more effort to major pests when implementing pest management strategies. The priorities may be determined by the worldwide economic losses posed by the individual type of pest. For instance, whiteflies have been reported in Central America, North America, South America, China, Central Africa, South Africa, South East Asia, Pakistan, and India [70,71]. Similarly, cotton mealybugs have been reported in more than 26 countries in various ecological zones [72]. Boll weevils have been reported throughout southern Texas to Argentina [73]. Several studies have indicated a tremendous increase in serious destructive pests within the cotton ecosystem. For example, in the USA, the cotton fleahopper was ranked ninth in early 1999 and fourth or sixth in 2007 in the last decade [74]. The present study highlights the major pests and corresponding yield losses reported in different studies (Figure 5). The magnitude of yield losses in cotton yield may differ based on the local production practice, the type of pests, pest population, the stage of the crop, and pest-supporting conditions in given ecological zones of a specific region. For example, depending on the stage of the crop and the infestation intensity in the field, fall armyworm larvae may cause yield losses of 25.8 to 100% [75,76]. Given the maximum losses likely attained by the most destructive pests, cotton farmers may lose even more than 50% of their yield when attacked by such major pests. In case of an attack by a major pest such as a boll weevil, farmers may apply up to fifteen (15) insecticide sprays per season because of clear decisions or guidance about the insect population threshold estimation [76,77]. Unfortunately, managing major cotton pests is complex as most farmers and advisors use informal knowledge to control the dynamics of such pests [78,79,80,81,82,83]. The recent shift from deep fallowing/plowing to reduced or no-till farming practices has also been linked to severe pest prevalence and attack consistency [84]. The severe pest attack was associated with fewer farmers (approx. 8.33%) engaging in traps for the mass trapping of pests [85]. Farmers have repeatedly been unable to control the severe pest attack because of the pest’s ability to migrate from multiple crop hosts to cotton and a temperature that favors pest reproduction [86]. Preventive seedling protection has been recommended as one of the main approaches to reduce cotton infestations and encounter the overuse of pesticides in the case of unknown pests. However, this approach may adversely affect nontarget insects, including honey and other pollinators [87,88]. Creating a database of pest images and linking it with artificial intelligent models or sensor-based systems could easily classify multiple pests and recommend insect-specific insecticides to reduce nontarget insects’ sublethal adverse effects.

## 10. Recommendations for Future Research

The studies investigated here focused on detecting cotton pests and beneficial insects (predators and parasitoids) based on the images from the stems, undersides of leaves, the root of the bloom, the external or internal part of the bloom, and roots are still limited. Our review uncovers a scarcity of studies on detecting the insects that may harm plants, which spend a portion of their life or growth cycles in the soil. In addition, identifying pests from images with a complex background, concentrated problems, and similar-looking species in one image is a major challenge when identifying cotton pests. Based on this review, more studies should concentrate on possible attractants for such pest species and utilize the sticky board to capture them for imaging in the sensor region.

Attention should be given to some insects that may not have a long flight range, may not fly at all, or, as mentioned, spend a portion of their life cycles underground.

Moreover, most artificial intelligence techniques have employed CNN models to detect and classify cotton pests and beneficial insects. Nevertheless, using a typical CNN classifier, on the other hand, may need a large quantity of training and validation data, necessitating more time, more significant research expenditures, and compelling operational hardware. To identify cotton pests with little data, training time, and low-cost hardware, researchers should apply more efforts to understand the potential of Small CNN architectures such as AlexNet, GoogLeNet, Inceptionv3, SqueezeNet, ResNet-101, VGG16, ShuffleNet, InceptionResnetv2, MobileNetv2, and DenseNet201. Due to their cheap storage needs, fast training times, and excellent accuracy, such techniques should be combined with the utilization of ShuffleNet, SqueezeNet, and MobileNets. In addition, there have been inadequate pest detection, classification, and counting investigations considering abiotic factors. Some insect pests may exhibit unique behaviors depending on abiotic elements and influencing artificial intelligence performance. Consequently, additional research into these abiotic aspects is needed. Many studies identified the pests but did not further estimate the insect population dynamics.

## 11. Conclusions

So far, AI models and IoT sensor-based techniques can identify, classify, and count cotton insect pests or beneficial insects with reasonable accuracy. However, the main challenges were the insect location (on plant leaves, stems, holes in soils), obtaining enough data, identifying pests/beneficial insects from images with concentrated pests and similar-looking species, or obtaining insects’ acoustic signatures containing noise. On the other hand, IoT was limited by data storage, field remoteness, and a lack of defined optimum distance between the sensors when targeting pests or beneficial insects to estimate populations. Enhanced CNN algorithms, sticky boards, low-bandwidth devices, and more research on insect-specific attractants to bring them to the sensor region may overcome the mentioned challenges and improve AI and IoT in cotton production. In addition, our analysis found that few studies on AI and IoT explicitly address cotton pest predators/parasitoids and immature insects. Overall, this review has identified the current pest classes already detected by AI, pest recognition features, common variables, methods, intelligent systems, success, and challenges when identifying, classifying, and counting pests or beneficial insects in cotton. By combining these aspects, this review has contributed to the art of AI and IoT by identifying crucial elements in the design and development of future devices to easily monitor and detect pests/beneficial insects in cotton production.

## Figures and Tables

**Table 1:** Essential techniques for the evaluation of morphological variations between insect species during feature classifications.

S/No.	Morphological Insect Feature	Formula
1	Form Factor	=(4 × π × Area)/(Perimeter)^2^
2	Roundness	=(4 × Area)/(π × Max Diameter^2^)
3	Aspect ratio	=(Max Diameter)/(Mean Diameter)
4	Compactness	=(Sqrt ((4/π) × Area)/Max Diameter)
5	Extent	=Net area/Bounding rectangle

Source [21].

**Table 2:** Common techniques to measure the performance of artificial intelligent models during image processing.

No.	Analysis Term	Formula	Description
1	Accuracy (%)	=[(TP + TN)/(TP + FP + FN + TN)] × 100	Estimates the percentage of correct predictions made by a model
2	Precision	=[TP/(TP + FP)]	Indicates the quality of a positive prediction made by the model
3	Recall (sensitivity)	=[TP/(TP + FN)]	Evaluates how accurately the model is capable of identifying the relevant data
4	F1-score	=2/[(Recall)^−1^ + (Precision)^−1^]	Calculates the model’s overall accuracy by combining the precision and recall metrics in a twofold ratio.
5	Mean Average Precision	=(($\overset{n}{\underset{q=1}{\sum }}\mathrm{AP}(q))$/Q) × 100%	Shows the Average Precision metric obtained from Precision and Recall

True Positive (TP), True Negative (TN), False Positive (FP), False Negative (FN), Total pest categories (Q), and the average accuracy rate of results detected in each pest category AP (q) [11,26].

**Table 3:** Summary of recently detected cotton pests using AI.

Detected Insect Pests	Reference
Boll weevil, Cotton aphid, Cotton bollworm (larva), Cotton bollworm (adult), tobacco budworm (larva), Tobacco budworm S (adult), Soybean looper, Fall armyworm (larva), Fall armyworm (adult), Cotton leafworm, Cotton whitefly, Cotton bug, Pink bollworm, southern armyworm, and red spider mite	[1]
Cotton aphids, Flea beetles, Flax budworms, and Red spider mites	[11]
Mexican cotton boll weevil, Fall armyworm, Cotton bollworm, Cotton aphid, Cotton whitefly, Green stink bug, Neotropical brown stink bug, Soybean looper	[14]
Assassin Bug, Three-Corned Alfalfa Hopper, and Convergent lady beetle	[21]
Red spider mites and Leaf miner	[27]
Pink and American bollworms	[28,29]
American bollworm, Ash weevil, Blossom thrips, Brown cotton moth, Brown soft scale, Brown-spotted locust, Cotton aphid, Cotton leaf roller, Cotton leafhopper, Cotton looper, Cotton stem weevil, Cotton whitefly, Cream drab, Cutworm, Darth maul moth imago, Darth maul moth, Desert locust, Dusky cotton bug, Giant red bug, Golden twin spot tomato looper, Green stink bug, Grey mealybug, Hermolaus, Latania scale, Madeira mealybug, Mango mealybug, Megapulvinaria, Cotton stainers, Menida, Menida-versicolor, White-spotted flea beetle, *Myllocerus-subfasciatus*, Sri Lankan weevil, Painted bug, Pink bollworm, Brown-winged green bug, *Poppiocapsidea*, Red-banded shield bug, Red cotton bug, Red hairy caterpillar, Solenopsis mealybug, Spherical mealybug, Spotted bollworm imago, Spotted bollworm, Tobacco caterpillar Tomentosa, Transverse moth, Tussock caterpillar, and Yellow cotton scale	[29]
Cotton whitefly	[1,14,23,29,30,31,32]

**Table 4:** Challenges of artificial intelligent field devices, sources, and proposed solutions.

Challenge	Source	Solution
Power consumption	- High-power requirements of networking devices, micro-controllers, and embedded computers [58,59]	- Scheduling tasks, creating intelligent software with the fewest computations possible, and making idle mode. - Low data rates may be achieved with good energy economy when short-range communication is used at transmission distances of less than 20 m [59] - Based on the installation site and operation goal, IoT sensor nodes can be set up as reduced-function gadgets that only talk to full-function devices. - Convey data to the control center from intermediate nodes that receive it from other IoT nodes [59] - Solar power and other alternative energy sources [59,60] - Predicting power usage with energy forecasting devices and models
Failure to execute software	- Limitation of software and poor processing power in case of rapid data processing over wide range of sensor data sources [59,61]	- Comprehensive software testing before deploying
Service expiry fault	- Applications failing because of expired or terminated cloud services [62]	- Payment renewal of application services such as internet bundle and app services should be configured to the automatic payment mode
Network faults	- Network breakdowns, packet loss or corruption, congestion, or a problem with the destination node [61,62] - Inadequate Internet coverage causing connection issues that result in lost or incorrectly sent data [63]	- The design should include fault tolerance [61,62,64] - The design should include retrying failed packages and real-time reporting [65]
Security	- Security for devices and data privacy [61]	- Access control, authentication, and authorization techniques [66] - Make data encryption before transmission
Physical faults of hardware	- Issues with sensors, processors, memory, storage, and power supply [61]	- The device should be calibrated and undergo robustness testing before deployment [67]
Data cost	- High cost of transmitting images	- Reduce the frequency of sending images
Changes in environmental conditions	- Extreme weather conditions such as rain, extremely hot or low temperatures, wind, and other extreme situations	- Add weather check sensors and modules [68,69]

## Data Availability

Not applicable.

## Nomenclature

CNN	Convolutional Neural Networks
SVM	Support Vector Machine
KNN	K-Nearest Neighbors
ANN	Artificial Neural networks
RNN	Recurrent Neural Networks
DBN	Deep Belief Network
DBM	Deep Boltzmann Machine
LBP-SVM	Local Binary Patterns with Support Vector Machine
Faster R-CNN	Faster Recurrent Convolution Neural Network
ResNet	Deep Residual Network
DCNN	Deep Convolutional Neural Network
HD-CNN	Hierarchical Deep Convolutional Neural Network
SegNet	Semantic Segmentation Network
SSD MobileNet	Single Shot Multi-Box Detector MobileNet
BP Neural Network	Back Propagation Neural Networks
Bi-Directional RNN	Bi-directional Recurrent Neural Network
LSTM	Long Short-Term Memory
GNSS	Geographical Navigation Satellite Systems
